# Subcutaneous Injections of Strychnine

**Published:** 1893-09-30

**Authors:** 


### New Drugs and Preparations.

[We should be glad to receive, at our Office, 428, Strand, London, W.C., from the manufacturers, specimens of all new preparations and drugs which may be brought out from time to time.]

### SUBCUTANEOUS INJECTIONS OF STRYCHNINE.

The therapeutical uses of strychnine may be briefly stated to be three in number, namely, (1) as an aid to digestion, (2) as a general tonic, and (3) as a means of increasing muscular tone and reflex nervous excitability in cases of severe depression. The first two of these uses may be dismissed without further comment; the fact that the tincture of nux vomica is said to be the most widely used drug in the pharmacopœia being sufficient evidence of the popularity of its active ingredient. It is in the third group of cases that the beneficial effects of hypodermic injections of strychnine are most typically seen.

In this connection two sets of cases may be considered, namely, (1) those in which death from exhaustion or general failure of vitality is imminent, and (2) those in which certain paralyses of nervous or muscular origin are present.

A typical example of the first of these two classes is to be found in the late stage of a severe case of pneumonia, in which there is not only great exhaustion and failure of vital power, but also depression and loss of excitability of the respiratory nervous centre. In such a case the vigorous and judicious administration of strychnine will sometimes keep the patient alive until his crisis, and thus ultimately save his life. One or two points are to be observed. In the first place a far more powerfully stimulating effect can be obtained by the subcutaneous injection of the drug, than by its administration by the mouth, and larger quantities can be given in this way than would otherwise probably be tolerated by the stomach. Secondly, the doses must be large and frequently repeated. The actual amount must, of course, be decided in each case by the age and condition of the patient, but injections of ♏iii.—v. of the liquor strychninæ repeated at intervals of a few hours each, will usually be well borne, and the writer has even seen ♏x. given every three hours for several doses without producing any unpleasant symptoms. Thirdly, when once this treatment has been adopted, it must be steadily continued at regular intervals during both night and day, great, and perhaps even fatal, depression following its sudden cessation when once the full effect of the drug has been established. It is thus a somewhat difficult method of treatment to carry out, unless a reliable nurse is in charge of the case. Fourthly, the drug must be diluted with three or four times its bulk of water, and a strictly antisepticised needle used; otherwise irritation and inflammation of the skin is likely to arise at the seat of puncture.

In typhoid fever, similar good results will sometimes follow the same method of treatment, especially in those cases in which the disease has nearly or quite run its course, but the patient appears likely to die from extreme exhaustion, and in which the ordinary methods of stimulation have ceased to produce any appreciable effect. As an example, the writer may quote a case, recently under his care, in which the patient was apparently again and again saved from imminent death by the injection of ♏iv. doses of strychnine repeated every four hours night and day for several days. Here, however, the probable necessity for continuing the treatment, when once begun, for a longer period than is usually necessary in cases of pneumonia, must be carefully considered.

A final example of the use of strychnine injections, for the purpose of stimulation, may be found in those cases of mitral disease in which attacks of heart failure periodically occur, and which are probably more efficiently treated in this manner than by the administration of the ordinary alcoholic or ethereal stimulants.

Turning now to cases of paralysis of muscular or nervous origin, extremely good results will often arise from the long continued injection of sufficiently large doses of strychnine, always provided that only such cases are chosen as are from their nature capable of recovery or great improvement. It will thus be useless to adopt such treatment in most cases of paralysis of spinal origin, whilst cases of neuritis will usually afford the most striking instances of success. Here, again, large doses must be used, and it will generally be found that injections of as much as ♏x. each, repeated twice a day, will give rise to no inconvenience of any sort so long as the before-mentioned precautions with regard to dilution of the drug and sterilisation of the syringe are observed. The writer has recently seen a bad case of alcoholic neuritis in which such treatment, combined with the vigorous use of massage and galvanism, resulted in almost complete recovery of muscular power in the space of five weeks—a very unusually rapid improvement. In such cases the administration of strychnine by the mouth appears to have very little effect, even if larger doses are given than will usually be borne by the stomach.

The contra-indications for strychnine are few, and may be summarised by saying that the drug must be avoided in those cases in which there is any undue irritability of the nervous centres. Thus it would probably do more harm than good in the case of epileptic patients, and is certainly contra-indicated in cases of paralysis in which descending degeneration of the lateral columns has given rise to increased reflexes and aspastic condition of the muscles.

